# Limited Potential or Unfavorable Manipulations? Strategies Toward Efficient Mesenchymal Stem/Stromal Cell Applications

**DOI:** 10.3389/fcell.2020.00316

**Published:** 2020-05-19

**Authors:** Antonina Lavrentieva, Andrea Hoffmann, Cornelia Lee-Thedieck

**Affiliations:** ^1^Institute of Technical Chemistry, Leibniz University Hannover, Hanover, Germany; ^2^Department of Orthopaedic Surgery, Graded Implants and Regenerative Strategies, Hannover Medical School, Hanover, Germany; ^3^Institute of Cell Biology and Biophysics, Leibniz University Hannover, Hanover, Germany

**Keywords:** mesenchymal stem/stromal cell, donor variability, expansion protocols, bioreactor, potency

## Abstract

Despite almost 50 years of research and over 20 years of preclinical and clinical studies, the question of curative potential of mesenchymal stem/stromal cells (MSCs) is still widely discussed in the scientific community. Non-reproducible treatment outcomes or even absence of treatment effects in comparison to control groups challenges the potential of these cells for routine application both in tissue engineering and in regenerative medicine. One of the reasons of such outcomes is non-standardized and often disadvantageous *ex vivo* manipulation of MSCs prior therapy. In most cases, clinically relevant cell numbers for MSC-based therapies can be only obtained by *in vitro* expansion of isolated cells. In this mini review, we will discuss point by point possible pitfalls in the production of human MSCs for cell therapies, without consideration of material-based applications. Starting with cell source, choice of donor and recipient, as well as isolation methods, we will then discuss existing expansion protocols (two-/three-dimensional cultivation, basal medium, medium supplements, static/dynamic conditions, and hypoxic/normoxic conditions) and influence of these strategies on the cell functionality after implantation. The role of potency assays will also be addressed. The final aim of this mini review is to illustrate the heterogeneity of current strategies for gaining MSCs for clinical applications with their strengths and weaknesses. Only a careful consideration and standardization of all pretreatment processes/methods for the different applications of MSCs will ensure robust and reproducible performance of these cell populations in the different experimental and clinical settings.

## Introduction

**FIGURE 1:**
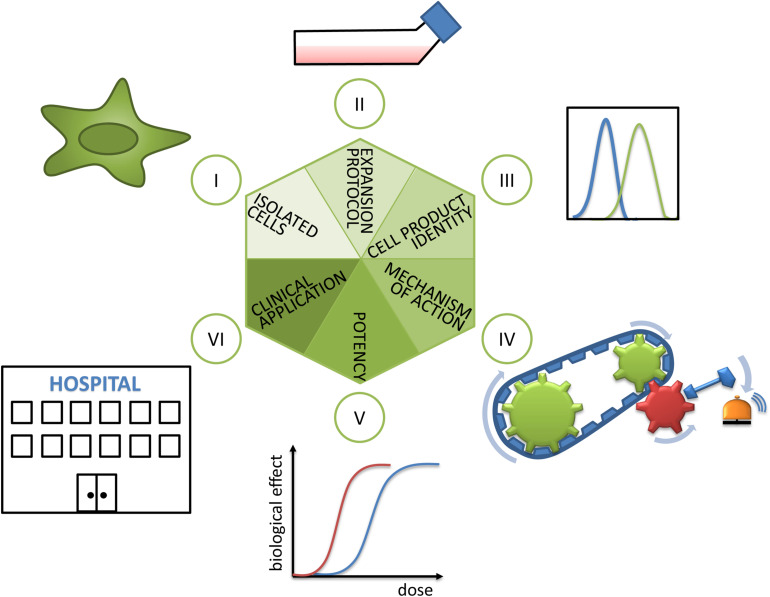
Hexagon toward translating MSCs’ promise into clinical reality. (I) As a first step, the isolated cells’ identity has to be analyzed. Here, the cell source in terms of donor and tissue of origin, as well as population heterogeneity, plays an important role and is discussed in sections “Cell Source: Adult Tissues or Birth-Associated Tissues”, “Choice of Donor and Recipient”, “Isolation Methods”, and “Biological Properties of MSCs *in vivo*”. (II) The decisive role of expansion protocols and (III) cell product identity for preparing MSC products for clinical use are explained in section “Expansion Protocols and Culture Conditions”. (IV) The importance of revealing mechanisms of action and (V) choice of valid potency assays or matrix of assays are detailed in section “Potency Assays”. (VI) Finally, during clinical application the selection of recipients (reviewed in section “Choice of Donor and Recipient”) and measurement of clinical effects have to be considered.

Mesenchymal stem/stromal cells (MSCs) have the capacity to differentiate into cells and tissues of one germ layer, here the mesodermal lineage, and are consequently multipotent. MSCs also secrete a variety of soluble factors and exosomes and, via contact with host cells, modulate functions of effector cells (Kabat et al., 2020). These features endow them with immunomodulatory, tissue-engrafting, cell-empowering (Najar et al., 2018), migratory, and homing properties. Despite a large history of research and use in clinical trials including some successful and spectacular examples based on either their differentiating capacities (Horwitz et al., 1999; Horwitz et al., 2002) or their secretory properties (Le Blanc et al., 2004) and nicely summarized in a plethora of reviews (e.g., Ballini et al., 2018 or Kabat et al., 2020), the understanding of MSC biology, their mechanism of action (MoA) in different biological contexts, and their targeted and routine use in the clinics is limited (Hoogduijn and Lombardo, 2019). In this mini review, we propose a hexagon of steps to consider during selection, pretreatment, analysis, and application of MSCs in order to improve the transferability of promising preclinical results into clinical success (Figure 1). In the following, the six steps will be discussed in detail.

## Cell Source: Adult Tissues or Birth-Associated Tissues

**FIGURE 2:**
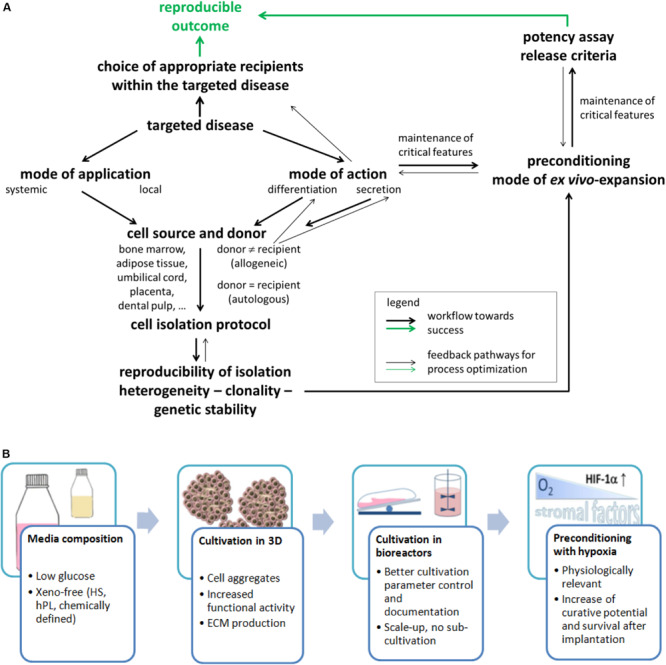
**(A)** Flowchart of important stages for resolving the challenges on the way toward efficient MSC applications. This will need to consider several important issues that are depicted in the present figure. This will also need a constant reiterative optimization of different aspects compared to the current state of the art. Such a course of action will finally allow enhanced matching of *in vitro* and *in vivo* data and ultimately an enhanced translation of data from laboratory investigations into clinical practice through a reproducible and predictable outcome. **(B)** Important factors and expansion conditions to consider for improving the final MSC product quality.

Bone marrow (BM) from animals was the protagonist tissue of origin in the 1960s when these cells were first identified (Friedenstein et al., 1968). Meanwhile, MSCs are isolated and expanded from a number of tissues from adult human donors (BM, adipose and dental tissue, muscle, and skin) and from birth-associated human tissues (placenta, amnion, Wharton jelly of the umbilical cord, or umbilical cord blood). Birth-associated tissues offer the advantage of non-invasive acquisition; the cells are in a developmentally early state and have higher immunosuppressive activity (Haase et al., 2009; Deuse et al., 2011; Hass et al., 2011). Despite similarities in morphology, immunophenotype with respect to selected cell surface antigens [while others depend on the tissue source (Lv et al., 2014)] and differentiation *in vitro*, MSCs sourced from distinct tissues may have a different developmental origin (Bosch et al., 2012) and do not necessarily have equivalent biological properties (Reinisch et al., 2015; Sacchetti et al., 2016). This is illustrated by several examples: (i) It was shown that MSC populations from different tissues differed widely in their *in vivo* differentiation potential and transcriptomic signature (Sacchetti et al., 2016). (ii) HLA class I expression was significantly reduced in human amnion MSCs compared to MSCs from BM until passage 6 (Pogozhykh et al., 2015). This indicates that the immunomodulatory and immunoevasive properties of MSCs (Ankrum et al., 2014) from different tissue sources may vary. (iii) Clinical studies using MSCs from BM were considered to be safe even with systemic application by infusion. However, because of the higher expression of tissue factor (also called CD142) on MSCs from adipose or birth-associated tissue compared to MSCs from BM, there is a notably increased risk for incompatibility with blood during intravascular application, caused by the instant blood-mediated inflammatory reaction (IBMIR). This leads to thrombotic complications and reduced engraftment (Moll et al., 2019). In summary, the intended mode of application (systemic or local, cell suspension, or mixed with a carrier system) and MoA of the cells (e. g differentiation into a desired cell type or secretion for immunomodulation) from different sources need to be carefully considered and compared for the choice of tissue source as indicated by forward and backward arrows in Figure 2A.

## Choice of Donor and Recipient

Isolation and expansion of MSCs from human BM were reported in 1992, and in 1999, these cells were administered into human patients (Horwitz et al., 1999). Since that time, as well autologous as allogeneic applications have shown success, with most studies using allogeneic cells (Pittenger et al., 2019). Such allogeneic use is possible because MSCs are considered to be immune evasive (Ankrum et al., 2014). Autologous cells may be an attractive option, available even from perinatal tissue when cryostored—here, however, the system of cryobanks needs to be expanded (Bieback and Brinkmann, 2010; Brown et al., 2019; Kamal and Kassem, 2020). However, the prerequisite for use of autologous cells is that they are not affected by the disease to be treated or by comorbidities. Only an allogeneic setting offers the option to select for cell populations with particular properties (arrows in Figure 2A). This choice, however, also depends on the tissue source for cell retrieval. In a proinflammatory environment, the immunosuppressive activity of MSCs is affected with low doses of inflammatory cytokines inducing an immunostimulating phenotype but high doses inducing an immunosuppressive phenotype as demonstrated in a number of studies, e.g., reviewed in Najar et al. (2018). Consequently, the recipients/patients and their disease to be treated may become a decisive factor for success of MSC-based therapies (Martin et al., 2019). Figure 2A summarizes some important points.

## Isolation Methods

In the case of a fluid tissue such as BM, mononuclear cells are used directly or purified by density gradient centrifugation and plated at defined (clonal or non-clonal) or non-defined cell density. In the case of solid tissues, explant cultures or enzymatic digestion are used (Hoffmann et al., 2017). MSCs are subsequently identified as compact colonies containing spindle-shaped cells. The first passage is usually performed by detaching the cells with a protease once individual clones have reached a certain size as defined by the individual scientist. Although macrophages also grow in a plastic-adherent manner, they do not persist in the cultures as demonstrated by the absence of expression of antigens such as CD11b, CD13, and CD163 (e.g., Schack et al., 2013). Histological investigations with spatial resolution of tissue or single-cell analyses by flow cytometry revealed different subpopulations in different microanatomic sites, even for BM as a single tissue (Rasini et al., 2013) or resulted in isolation of selected subpopulations [CD271: (Kuci et al., 2013), STRO-1: (Shi and Gronthos, 2003)].

Multicolor lentiviral barcode labeling was applied to follow the clonal dynamics of *in vitro* MSC isolation and expansion from pieces of umbilical cord (Selich et al., 2016). MSCs migrating out of the tissue pieces during explant culture initially demonstrated a highly complex mixture of different cell clones. However, with time, a massive reduction in abundance of clones was detected. This led to a preference for only few cell clones within few passages that are necessary to generate clinically relevant cell numbers. Also interesting, initiating novel MSC cultures from the same piece of tissue revealed the existence of more primitive cells as evidenced by a stronger secretion of cytokines after stimulation (Selich et al., 2019). It seems highly likely that similar observations would be found not only for MSCs isolated from solid tissues but also for MSCs from a fluid tissue as BM. Such clonality needs to be considered in the future development of refined cell isolation protocols (Figure 2A) as they may result, for example, from more research into the biological properties of these cells *in vivo*.

## Biological Properties of MSCs *in vivo*

The straightforward preparation of MSCs *in vitro* has enabled an incredible amount of studies. However, the *in vivo* identity and biology are less clear and less well characterized. Researchers identified CD146-positive cells in BM as adventitial reticular cells in the subendothelial layer of sinusoids (Sacchetti et al., 2007) and as the *in vivo* equivalent of *in vitro* MSCs. Self-renewing capacity of CD146-expressing cells as a characteristic of genuine stem cells was demonstrated by secondary passage (Sacchetti et al., 2007) and serial transplantation (Serafini et al., 2014). Independent results demonstrated that MSCs apparently can derive from pericytes (CD34^–^CD146^+^) and from adventitial cells (CD34^+^CD146^–^), and they termed them perivascular stromal/stem cells (Corselli et al., 2013). In September 2018, a consortium identified “the human skeletal stem cell” as a self-renewing, multipotent stem cell entity (Chan et al., 2018; Ambrosi et al., 2019). This cell was characterized by surface expression of podoplanin, CD73 (ecto-5’-nucleotidase), and CD164 (Endolyn). It is important to state that most of these studies avoided long-term *in vitro* expansion of their isolated cell populations. From these studies, the possibility emerges that there are several populations of “skeletal stem cells,” which await further identification and characterization, in particular with respect to their MoA and potency.

## Expansion Protocols and Culture Conditions

Recent reviews on the clinical development of MSCs highlighted the importance of *ex vivo* MSCs’ manipulation (Guadix et al., 2019; Mastrolia et al., 2019; Yuan et al., 2019). *Ex vivo* expansion and preconditioning are considered crucial for cell functionality after implantation. Because the MoA in the treatment of different diseases is not exactly known, it is important to maintain all possible initial MSC functions, including retention of all receptors (to receive external stimuli) and adhesion molecules (for migration, homing, and interaction with other cells), as well as production of cytokines, chemokines, growth factors, and extracellular vesicles (for stromal function). In the following, some factors and conditions that are considered to be important for the final cell product quality are reviewed (Figure 2B).

### Media Composition: Basal Medium and Supplements

Although long-term (over 40 days) expansion has a negative impact on migration, differentiation, genetic stability, and proliferation of MSCs (Wagner et al., 2009; Hladik et al., 2019), rapid expansion does not guarantee the quality of the cell-based products. Application of high-glucose medium for expansion is considered to enable fast cell growth by the easy availability of glucose (Nuschke et al., 2016); however, glucose concentrations greater than 1 g/L also lead to cellular senescence (Zhang et al., 2017), including telomere shortening and genomic instability (Parsch et al., 2004). It was demonstrated for BM-MSCs that low-glucose Minimum Essential Medium Eagle Alpha Modification (1 g/L glucose) is the better medium choice compared to Dulbecco’s Modified Eagle’s Medium and Iscove’s Modified Dulbecco’s Media (Sotiropoulou et al., 2006). In many studies, MSCs were expanded using fetal calf serum (FCS), whereas nowadays the use of FCS for MSC expansion is not favored anymore, because besides the ethical issues of FCS production (collecting the serum from unborn calves), viral, mycoplasm, or prion infections can be transferred to the patient (Bieback, 2013; Jonsdottir-Buch et al., 2013; Hemeda et al., 2014; Mastrolia et al., 2019). Moreover, animal xenogenic compounds (proteins and polysaccharides) from FCS are internalized by the cells and can cause immune response after MSCs implantation, even if autologous cells were used (Spees et al., 2004). As an alternative, human serum or human platelet lysate can be used for MSCs’ expansion (Mannello and Tonti, 2007; Bieback, 2013; Hemeda et al., 2014). Chemically defined xeno-free media could provide acceptable cell growth and elimination of the risk of carrying over pathogens (Spees et al., 2004), but the evaluation of functional characteristics of the cells cultivated in such media has to be improved (Lee et al., 2017).

### Cultivation in Bioreactors

Traditionally, anchorage-dependent MSCs are expanded in static multiple planar T-flasks or multilayered flasks. Such “open systems” provide only a limited surface area and little control over cultivation parameters, are labor-intensive, and can lead to a high contamination risk and impaired cell function (Bunpetch et al., 2017; Mizukami and Swiech, 2018). In contrast, expansion in bioreactors (“closed systems”) provides higher cell yield, full control, and documentation of cultivation parameters, as well as better spatial distribution of nutrients, pH, and oxygen (Bunpetch et al., 2017). Several bioreactor types are used for MSCs’ expansion: rotating-bed bioreactors (Neumann et al., 2014), stirred tanks (Sart and Agathos, 2016; Mizukami et al., 2019), bag reactors (Das et al., 2019), hollow fiber (Mennan et al., 2019), and fixed bed reactors (Weber et al., 2010; Osiecki et al., 2015). In the case of stirred tanks or bags, anchorage-dependent MSCs are cultivated as aggregates or on microcarriers (Alimperti et al., 2014; de Soure et al., 2016).

### Cultivation in Three-Dimensional Systems and Under Dynamic Conditions

A growing number of publications demonstrate that cultivation/expansion of MSCs in three-dimensional (3D) systems increases their functional activities in terms of angiogenic (Cheng et al., 2013), anti-inflammatory (Bartosh et al., 2010), and immunomodulatory (Noronha et al., 2019) properties. The most common method for 3D MSC cultivation are cell aggregates, generated by hanging drops, ultralow adhesion plates, centrifugation, and structured microwells (Bartosh et al., 2010; de Soure et al., 2016; Egger et al., 2018). To allow better nutrient transport, aggregated cells are then cultivated under dynamic conditions: agitation, stirring, or perfusion. It is important to note that cultivation in 3D aggregates allows MSC expansion in serum-free conditions (Alimperti et al., 2014).

### Preconditioning With Hypoxia

Several preconditioning strategies (also called “priming”) were developed for MSCs (Hu and Li, 2018; Noronha et al., 2019; Castilla-Casadiego et al., 2020). These strategies include the use of pharmacological or chemical substances, small molecules, cytokines, physical factors, biomaterials, and hypoxia. Here, we focus on priming with hypoxia as it represents a complex, multilevel, and physiologically relevant strategy. Preconditioning of MSCs in hypoxia triggers (via the stabilization of Hypoxia Inducible Factor (HIF)-1α and other adaptation mechanisms) an upregulation of various functions and does not only help MSCs to survive after implantation, but also increases their curative/stromal potential. Exact details of hypoxic treatment protocols are still under discussion: oxygen concentration, duration of preconditioning, MSCs’ isolation under hypoxia, and reoxygenation. Oxygen concentration (1–5% O_2_) should be low enough to trigger adaptation, but not too low as to cause apoptosis (El-Badawy et al., 2016). Serum deprivation should be avoided during hypoxic conditions, because it also leads to apoptosis (Chen et al., 2018). MSCs isolated from different sources demonstrated higher proliferation and migration rates, metabolic activity, cytokine, and receptor expression, as well as improved immunomodulatory properties and genetic stability under hypoxia (Lavrentieva et al., 2010; Estrada et al., 2012; Haque et al., 2013; Jiang et al., 2015; Choi et al., 2017; Fabian, 2019). Of note, hypoxic conditions should be preferred over using hypoxia-mimicking reagents (e.g., HIF stabilizers), because true hypoxic preconditioning can lead to the involvement of unknown, additional mechanisms beyond the HIF pathways (Pugh and Ratcliffe, 2017; Chakraborty et al., 2019).

## Potency Assays

While preclinical data on the efficacy of MSCs to treat pathological conditions are promising, translating this into clinical success is not straightforward. The cellular heterogeneity that is, on the one hand, an intrinsic property of cell communities—even in genetically identical cell populations (Wilson et al., 2019)—and on the other hand in case of clinically applied MSCs caused by the factors and parameters discussed above, might be one of the major reasons for the observed discrepancy in MSC efficacy (Galipeau and Sensebe, 2018). Despite these heterogeneities, how can we ensure that preclinical results hold true in clinical studies? The regulatory authorities reply to this question with the demand for potency assays. The European Medicines Agency EMA defines potency as “the measure of the biological activity using a suitably quantitative biological assay (also called potency assay or bioassay), based on the attribute of the product, which is linked to the relevant biological properties” (European Medicines Agency [EMA], 1999). Furthermore, “A correlation between the expected clinical response and the activity in the biological assay should be established in pharmacodynamic or clinical studies” (European Medicines Agency [EMA], 1999). In other words, potency assays measure the biological activity of a cellular product to ensure its intended function at a specific dose. Thus, the assay is meant to guarantee the comparability of different cellular products and of different lots of one product (Hematti, 2016).

The first prerequisite for defining a valid potency assay is to know the pathophysiology of the disease to be treated with MSCs and to know the MoA by which they exert their effects. The MoA of MSCs is highly dependent on the disease and microenvironmental tissue context in which they are applied. In most cases, the MoA of MSCs is complex involving not only direct but also indirect effects, e.g., via nearby cells. Roughly, the presumed MoA of MSCs can be subdivided into effects related to their following properties: (i) differentiation capacity, (ii) ability to engraft, and (iii) release of paracrine signals (Salvadori et al., 2019). Which capacity contributes to the potency of MSCs in different diseases to which extent is yet to be determined. The situation is further complicated by the phenomena of IBMIR (Moll et al., 2019) and efferocytosis (Galipeau and Sensebe, 2018).

The MoA is the link that is needed for the causal correlation of potency assays that measure a biological activity to the intended clinical response. In some diseases, the MoA might be traced back to one primary activity of MSCs. However, in most cases, MSCs’ action is a complex network of direct and indirect effects, and following only one effector pathway might lead to misinterpretable results. Therefore, the International Society for Cellular Therapy recommended developing matrix assay approaches that can cover the multiplicity of pathways involved in the MoA for a certain application (Galipeau et al., 2016). Wide and/or targeted OMICs approaches, particularly transcriptomic and metabolomic analyses, help identifying crucial factors in these networks that can be used as targets in the development of potency assays for MSCs in different diseases (Chinnadurai et al., 2018). Single-cell RNAseq of MSCs (Freeman et al., 2015) even opens the avenue for finding such targets in stemness-related effects that are transmitted by the small fraction of stem cells in the applied MSC populations.

In all cases, where the exact MoA is not fully solved, the dilemma of defining valid potency assays can only be solved by analyzing fundamentally and in detail which cells within the applied MSC population exert which effects in which way.

## Conclusion

In this mini review, we highlight some important points in the hexagon of steps (Figure 1) that are necessary to translate the promising results obtained with MSCs *in vitro* into successful cellular therapies. This includes as a first step ensuring the isolated cells’ identity, which is influenced by selection of donors and tissue sources as well as MSC population heterogeneity. In the next steps, optimized and standardized expansion protocols as well as guaranteed quality and identity of the produced cells are essential. At the same time, often unknown MoAs and choosing valid potency assays are currently major challenges in this regard. During the last step—clinical application—the choice of recipients is one decisive parameter. Making informed choices in each of these steps will contribute to improved matching of *in vitro* and *in vivo* data and ultimately an enhanced translation of data from laboratory investigations into clinical practice (Figure 2A).

## Author Contributions

All authors contributed equally to the conception, writing, and editing of the manuscript.

## Conflict of Interest

The authors declare that the research was conducted in the absence of any commercial or financial relationships that could be construed as a potential conflict of interest.
